# Medical communication skills training in the Indian setting: Need of the hour

**DOI:** 10.4103/0973-6247.75968

**Published:** 2011-01

**Authors:** Sanjoy Chatterjee, Nandita Choudhury

**Affiliations:** *Consultant Oncologist, Tata Medical Center, Kolkata, India*; 1 *Ex faculty, Nirma University, Ahmedabad; Highland Woods, Newtown, Kolkata, India*

**Keywords:** Medical communication skills, transfusion medicine, medical education, blood donor motivation

## Abstract

Advances in science and technology have revolutionized medical services in the last two decades. Medical education in the undergraduate and postgraduate courses has tried to keep pace with the changes and several curriculum modifications have taken effect. One of the commonly seen changes include active participation in “communication skills” training and implementation of the same in practice. This article discusses the practical issues one would face in day-to-day medical communication and highlights the necessity of the same in the Indian setting, with a focus on transfusion medicine.

## Introduction

A multidisciplinary approach is recommended in the management of most medical ailments. Communication between doctors, paramedical staff, and importantly, between the medical team and the patient and relatives has been discussed in the medical literature. Patients have different psycho-social needs and tailoring the communication to the patients’ requirements is highly valued.[1,2] Communicating the key points during each step of the patient’s journey is now considered to be an essential criterion for good medical practice and improves the job satisfaction of doctors.[3] The benefits of communicating appropriately have been investigated in the setting of clinical oncology and studies have reported improved treatment adherence and better psychological performance by our patients.[4] Studies[5] have also looked at cultural and gender differences in the motivation of blood donors, making it important to develop individualized communication strategies. Communication skills are not routinely taught in many Indian medical undergraduate or postgraduate courses, and there is a feeling that in a busy outpatient department, such skills may not be optimally applied. The article highlights some of the benefits of using appropriate communication skills and suggests a framework to train medical personnel.

The term communication is one of the most important and significant terms of the English language. It has its root in the Latin word *communicare*, which means to share, and this sharing is of information, knowledge and thoughts. Communication touches every sphere of our lives. Everything done throughout the day involves some or the other kind of communication – at work or at home, in politics, commerce, sport, entertainment, the financial world, education, and more specifically, medical education. With the advent of new and more sophisticated technologies, our world is fast shrinking into a global village. Therefore, the ability to communicate effectively, not only through verbal means but also non-verbally, has become very essential. While students feel the need to communicate successfully through oral and written media for their academic tasks, professionals face numerous challenges of communicating effectively and efficiently in their workplace. Lack of communication and the inability of people to communicate effectively causes a large amount of stress, frustration, anger, resentment, misunderstanding and disappointment.[6]

Education is all about communication - not only of hard facts but also of thoughts and ideas and proposals on which to base discussion and debate. However, there is one thing lacking in almost all education systems around the world and that is, teaching students how to communicate their thoughts to others. This results in producing professionals who may have good domain knowledge but are unprepared for what the world needs. This is particularly true in the field of medical education.

## The Indian Setting

The US Department of Health and Human Services has reported differences in health between different ethnic groups and in patients with limited English proficiency (LEP).[7] The patient populations in Indian outpatient clinics are diverse and there is a significant variation in their spoken language, cultural, educational and social background. The doctor is therefore required to individualize the information for every patient depending on the diverse backgrounds. We look at the different strategies that can be used to effectively convey this to our patients.

**Language and educational barriers:** It has been reported that patients with LEP in USA have had less referrals for preventive programs and had more diagnostic investigations. In India, the medium of medical education is English and the physician may not be adept with the patient’s spoken language, leading to similar shortcomings. Using trained interpreters to assist in such consultations has been recommended.[8] Although trained interpreters are available in many institutions in India, many still rely on the assistance of fellow doctors or the patient’s relatives to provide assistance and act as “untrained” interpreters. The need for structured training on the use of interpreters has not been widely established in many medical school curricula in the country, and with many interpreters still “untrained”, the value of a program to train medical students should be addressed as a matter of urgency. It must be noted that the benefits of using an interpreter are best derived once we have a program to train medical students in place.[9] The question about when to fit such a program in the medical curricula is debatable but a successful implementation earlier in the medical training could assist students in getting the most benefits as clinical involvement is now allowed quite early in the training. Similar justification was used by McEvoy *et al*. from the Albert Einstein College of Medicine, USA.[10] The structure of the program which spread over 3 years included small group discussions. In the first year, students were required to value and identify the importance of having an interpreter service. In the second year, they were asked to demonstrate the patients’ perspective and belief about the illness and importantly they were taught on how to use an “untrained” interpreter effectively. The final year was devised to address issues based on robust feedback from the faculty after the post sessions discussions in the previous year. The program had improved the competencies of the student in communicating with the patient and using untrained interpreters effectively. Such a program in the Indian setting could improve the patients’ clinical and psychological outcome.

**Cultural and social diversity:** Respecting the cultural issues of the patient can improve the confidence between the patient and doctor. Appropriate training into understanding the varied cultural requirements could help the doctor individualize the management of the patient. In India, there is a big cultural variation and it is required that the medical physician should be well aware of the majority of “do’s and don’ts” associated with the common cultural and ethnic backgrounds of the patient. These should be included in the communication skills training program and senior physicians must highlight the importance of such cultural sensitivities during ward rounds.[11]

## Communication in Transfusion Medicine

Transfusion medicine has undergone a sea change from a small dingy room in one corner of the clinical pathology department into a full-fledged medical speciality. Now we see well-designed blood banks with sophisticated equipment supported by the latest information technology available, and highly trained doctors, nurses, lab technologists and non-medical staff. Introduction of modern plastic bags in place of glass bottles, gamma ray sterilized disposable transfusion sets, optimum use of blood and its various components and modern laboratory technologies have resulted in changing the quality of blood banking and blood transfusion service. However, the one thing that has not changed since the dawn of blood transfusion of the modern era is the need of human volunteers to donate blood.[12] This is where effective communication skills are highly required. No transfusion service can exist without blood and there can be no blood without human blood donors. Blood donation is a highly sensitive topic since there are several misconceptions, myths, rumors and fears surrounding it, especially in a country like India. People generally are not self-motivated to donate blood and hence the need for persuasive and motivational communication. Awareness for regular and voluntary blood donation has to be improved to attain 100% voluntary donation. The need for blood donation has to be communicated in a persuasive, sustained and scientific manner in simple, listener-friendly language to the community at large.

Blood donor recruitment and retention are two areas in transfusion service that entail highly specific communication. In blood donation, we have first time donors and repeat donors. Highly persuasive communication skills are essential to motivate a person to donate blood. Once that person becomes a first time donor, effective communication will be required to make him/her a repeat donor. Blood transfusion service has two types of clients: the blood donors and the blood users (patients as well as clinicians). Therefore, training programs in communication skills have to be imparted to both technical and non-technical staff in a blood bank. The onus should be on the State Blood Transfusion Councils to initiate structured training programs in this sphere.

## Setting up a Communication Skills Training Program

Various models can be used which could enable adequate training to sharpen the skills required for communication. Based on a recent recommendation,[13] mandatory communication skills training courses should be made available in the undergraduate and postgraduate medical training. Whilst longer courses[9,10] within the undergraduate curriculum have been proven to be of benefit, not all courses have such modules. Simple areas like how to listen effectively, when to pause, how to allow patients to vent their frustration and how to encounter barriers of communication (like language and cultural issues) can be covered in a short workshop over a few days. A recent meta-analysis of 13 controlled studies of communication skills teaching has[14] shown a moderate improvement in the communication skills following attendance of such courses. Similar results have been reported by a prior Cochrane review group.[15] Following basic course, a further consolidation course was found to improve the skills of the participant further.[11] Due to the likeliness of fewer benefits from courses lasting for less than 3 days, the authors recommended that a training course for communication skills should be at least 3 days or more in duration to enable all topics to be covered in sufficient depth. Role plays with experienced facilitators who could guide the learners have been found to help in adequate development of the necessary skills, thereby implying that the skills of the trainers need to be adequately assessed.[16] Small group course discussions are preferred to ensure optimal participation by all the group members.[11] A set of objectives must be agreed upon prior to starting the course. Similarly, specific goals on how to handle emotionally difficult clinical scenarios, build relationship and confidence, and discuss complex information with the patient must be covered in these courses.[11] Various methods of teaching have been used, including the use of videos, role plays, and didactic lectures. A validated outcome measurement tool should be used to assess the benefits of such courses.

## Discussion

Rapid progress is being made worldwide to improve medical communication skills of health professionals. The idea of effectively communicating the risks and benefits of any procedure is immensely important. Even in simple community based program like “blood donation”, there appears to be a significant diversity on the basis of motivational elements associated with each gender. Informed consent for screening possible donors for HIV and Hepatitis B is required. These are socially and clinically complex issues which need to be addressed sensitively. Breaking bad news after a positive report from the above text must be done in line with the individual patients’ needs. Similar issues, sometimes more complex, are required to be dealt by the medical professional in other challenging clinical situations like oncology. This brings us to the question whether the medical professionals in India are adequately trained to provide the necessary service to the patient in a busy clinical setting. Can the professional cope with the emotional demands required to perform his or her role adequately?

We have tried to answer these questions in our article. There are now good quality data from meta-analyses,[12,13] which confirm the benefits of having a training program. It is therefore high time that we insist on having a structured communication skills training program in our undergraduate and postgraduate courses. Workshops to assist the medical team must also be organized in regional centers to ensure that high standards of patient care are achieved. Whilst most studies have shown improvements in communication skills of the medical staff[12,17] following such courses, an improvement in the emotional aspects and job satisfaction has also been reported. A small criticism of the available evidence is the fact that very few studies have objectively documented significant benefits for patients following counseling by a trained doctor. This area was highlighted in a recent consensus meeting[11] and future studies to confirm the effects have been recommended.

## Conclusion

The use of effective and formal communication skills training is required for medical professionals. The availability of such courses in our country is fragmented and a structured program needs to be in place to ensure that our doctors are supported adequately and patients get the best standards of care. Of course, transfusion medicine also involves non-medical personnel, but even they need to be properly trained to bring about 100% donation and improve the present standards of blood banking in India.
